# Determining the Target Concentration of Propofol for Sedation in Patients Undergoing Endoscopic Retrograde Cholangiopancreatography: A Target-Controlled Infusion Approach

**DOI:** 10.7759/cureus.62936

**Published:** 2024-06-22

**Authors:** Surbhi S, Udit Dhingra, Gaurav Sindwani, Anil Yadav, Vinod Arora, Deepak K Tempe

**Affiliations:** 1 Anesthesiology, Institute of Liver and Biliary Sciences, Delhi, IND; 2 Hepatology, Institute of Liver and Biliary Sciences, Delhi, IND

**Keywords:** bis, tci, endoscopic retrograde cholangiopancreatography (ercp), monitored anesthesia care, procedural sedation and analgesia

## Abstract

Introduction

Endoscopic retrograde cholangiopancreatography (ERCP) is vital for diagnosing and treating biliary and pancreatic diseases, necessitating deep sedation typically achieved through total intravenous anesthesia. Propofol, with its favorable pharmacokinetic profile, is the preferred sedative, but conventional administration methods of mg/kg boluses or infusion rates pose challenges. Target-controlled infusion (TCI) systems offer a solution that ensures precise dose delivery of propofol. Despite its widespread use, the literature lacks specific guidance on the target plasma concentration (Cp) of propofol for sedation in patients undergoing ERCP.

Methods

A prospective interventional study was conducted at the Institute of Liver and Biliary Sciences, Delhi, India to determine the target Cp of propofol for sedation during ERCP. The study enrolled 86 American Society of Anesthesiologists (ASA) grade I and II patients aged 18-70 years. The primary objective was to establish the optimal propofol concentration for sedation as guided by a bispectral index (BIS) value of 60-70. Secondary outcomes included induction time, recovery time, total propofol consumption, and the occurrence of adverse events (if any). The Marsh pharmacokinetic model guided the TCI pump, adjusting Cp until the target sedation was achieved.

Results

The mean Cp of propofol to maintain the BIS value 60-70 was 2.21 ± 0.42 µg/ml. Age-wise analysis revealed variations, emphasizing the need for individualized dosing. Induction time was 4.21 ± 0.68 minutes; recovery times were seven minutes (median, IQR: 5-10 minutes) for BIS >80 and seven minutes (median, IQR: 5-10 minutes) for achieving a Modified Observer’s Assessment of Alertness/Sedation score of ≥5. The mean propofol consumption was 6.24 mg/kg/hr. Side effects were minimal, with 1.16% experiencing transient hypoxia and hypotension.

Conclusion

The study establishes a mean target propofol concentration of 2.21 ± 0.42 µg/ml for sedation in ASA I and II patients undergoing ERCP.

## Introduction

Endoscopic retrograde cholangiopancreatography (ERCP) stands as the gold standard for diagnosing and treating biliary and pancreatic diseases, offering a less invasive alternative to open surgical or percutaneous procedures [1]. Despite its diagnostic prowess, ERCP is a complex and uncomfortable procedure requiring deep sedation, commonly achieved by total intravenous anesthesia (TIVA) using a sedative agent paired with a rapid and short-acting opioid [2].

Propofol, with its favorable pharmacokinetic and pharmacodynamic profile, emerges as the preferred sedative for ERCP [3]. However, the conventional methods of administering propofol, either through intermittent boluses or manual infusion pumps, pose challenges such as fluctuating plasma concentrations (Cps), hemodynamic instability, hypoxia, respiratory depression, inconvenience to the operator, and patient discomfort [4,5]. To address these issues, target-controlled infusion (TCI) systems offer a promising solution. TCI allows precise control over propofol delivery, maintaining optimal Cp to ensure effective sedation while minimizing side effects [6]. A target Cp is set, and the pump modifies the infusion automatically based on commonly employed pharmacokinetic models, such as Marsh, Schnider, Paedfusor, and Kataria, that guide the infusion pump to achieve and sustain the set Cp [7].

Despite the widespread use of propofol in TIVA for ERCP, the existing literature lacks specific guidance on the target concentration of propofol needed for sedation during this procedure. This prospective interventional study aims to bridge this gap, providing insight into the optimal propofol concentration for ERCP sedation using the bispectral index (BIS) as a depth of anesthesia monitor. The goal is to establish a balanced anesthesia care approach, optimize resource utilization without compromising intervention conditions, and minimize side effects to enhance patient safety and swift recovery.

## Materials and methods

This prospective single-arm interventional study was conducted between September 2023 and November 2023 at the Institute of Liver and Biliary Sciences, Delhi, India. Ethical approval was secured from the institute’s ethics committee (approval number IEC/2023/102/MA07), and the study was registered with the Clinical Trials Registry of India (CTRI/2023/09/057623), adhering to the principles outlined in the Declaration of Helsinki.

Patients falling within the American Society of Anesthesiologists (ASA) physical status I and II, aged between 18 and 70 years, scheduled to undergo ERCP, were enrolled after obtaining written informed consent. Exclusion criteria encompassed patients who declined consent, exhibited allergy to propofol or its constituents, were pregnant, had neurological or psychiatric disorders, had difficult airways, had liver cirrhosis, and had post-liver transplant status. The primary objective aimed to determine the target Cp of propofol necessary for achieving sedation (BIS 60-70) during ERCP. Secondary outcomes included calculating induction time, recovery time (stopping of infusion to BIS >80), time for clinical recovery (stopping of infusion to Modified Observer’s Assessment of Alertness/Sedation (MOAA/S) scale ≥5) [8], total propofol consumption, determining Cp in predetermined age groups (18-40 years, 41-64 years, and ≥65 years), correlation of age with propofol target Cp, and monitoring adverse events (hypotension, hypoxia, respiratory depression, bradycardia, tachycardia, patient movement, and pain due to drug injection), as well as nausea and vomiting.

In the ERCP suite, patients were positioned prone, and standard ASA monitors, along with BIS electrodes (BIS monitor, Covidien Medical, Boulder, United States), were applied. Baseline heart rate (HR), ECG, blood pressure, room air saturation (SPO2), and BIS values were recorded. Oxygen via nasal prongs was initiated at 4 liters/min. Patients received premedication with an injection of fentanyl 50 µg IV five minutes before the procedure, and intravenous plasmalyte solution was administered at 10 ml/kg/hour.

On the TCI pump (B. Braun Perfusor® Space target-controlled infusion, Melsungen, Germany), a 50-ml propofol (1%) filled syringe was attached, and patient demographic data were entered. The Marsh pharmacokinetic model was employed, with the initial target Cp set at 1.2 µg/ml based on the study by Imagawa et al., where they recommended 1.2 µg/ml as an ideal starting target Cp for propofol using the TCI pump [9]. The infusion was initiated, and Cp was increased by 0.2 µg/ml every two minutes until a BIS of 60-70 was achieved. Sedation levels were monitored using the responsiveness component of the MOAA/S score concurrently with BIS scores. Once the target sedation endpoint was achieved, the operator proceeded with endoscope insertion. A BIS range of 60-70 was maintained throughout the procedure. If the BIS value fell below 60 or exceeded 70 at any point, the Cp was decreased or increased by 0.2 µg/ml, respectively. Any patient movement was treated with a bolus of the induction agent, propofol 0.2 mg/kg. The Cp was noted every five minutes and was averaged to find the mean Cp.

Hemodynamic and respiratory parameters were monitored at specified intervals: on arrival (T0), when BIS 60-70 was achieved (T1), during scope insertion (T2), and at 5-minute intervals (T3 and so on) thereafter, any decrease in the mean arterial pressure (MAP) below 60 mm Hg or 20% below the baseline was treated with IV ephedrine 3-6 mg bolus, 0.6 mg atropine was administered when the HR decreased below 50 beats per minute, and for persistent tachycardia above 100 beats per minute lasting for five minutes, fentanyl bolus (0.5 µg/kg) was administered. If spontaneous ventilation was insufficient to maintain the SpO2 greater than 92%, the anesthesiologist gave a jaw thrust maneuver to assist, and on further desaturation, mask ventilation was instituted. After the procedure, infusion was stopped, and the duration of the procedure (from insertion of the scope to removal) and recovery time were noted. Patients were transferred to the post-anesthesia care unit after recovery.

Statistical analysis

The sample size was calculated based on findings by Imagawa et al. [9], who demonstrated that 80% of patients undergoing endoscopic submucosal dissection achieved a BIS range of 60-80 with propofol infusion by TCI at a Cp of less than 1.8 µg/ml and recommended 1.2 µg/ml as an ideal starting target Cp for propofol. Utilizing a 95% confidence interval, 10% relative error, and 10% contingency, a sample size of 86 was calculated. The data were recorded in Microsoft Excel (Microsoft Corporation, Redmond, United States). Continuous data normally distributed were presented as mean ± SD, while data not normally distributed were presented as median with IQR, and categorical data were expressed as numbers and percentages. Pearson’s correlation coefficient was used to find an age correlation with the target propofol concentration. A one-way ANOVA test was used to compare the mean Cp levels across the three age groups, and a p-value of less than 0.05 was considered statistically significant. All statistical analyses were conducted using IBM SPSS Statistics for Windows, Version 29.0 (Released 2022; IBM Corp., Armonk, NY, USA).

Definitions

A major adverse event was a need for endotracheal intubation, permanent neurologic impairment, or death. A transient hypoxic event was defined as a decrease in SpO2 <92% lasting for more than 10 seconds, which required airway intervention such as a chin-lift or jaw thrust maneuver, an increase in oxygen flow, or assisted ventilation. Adverse cardiovascular events included tachycardia (HR >100 beats/min), bradycardia (HR <50 beats/min), hypotension (MAP <60 mm Hg or a more than 20% decrease from the baseline), and arrhythmia (as any abnormal rhythm lasting for more than 10 seconds or requiring treatment). Induction time was defined as the time interval between the start of propofol administration and when a BIS value of 60-70 was recorded. Total procedure time was defined as the time from endoscope insertion to endoscope withdrawal. Recovery time was defined as the time to achieve BIS >80. The time to clinical recovery was defined as the time to reach a MOAA/S score of five after stopping the infusion.

## Results

**Figure 1:**
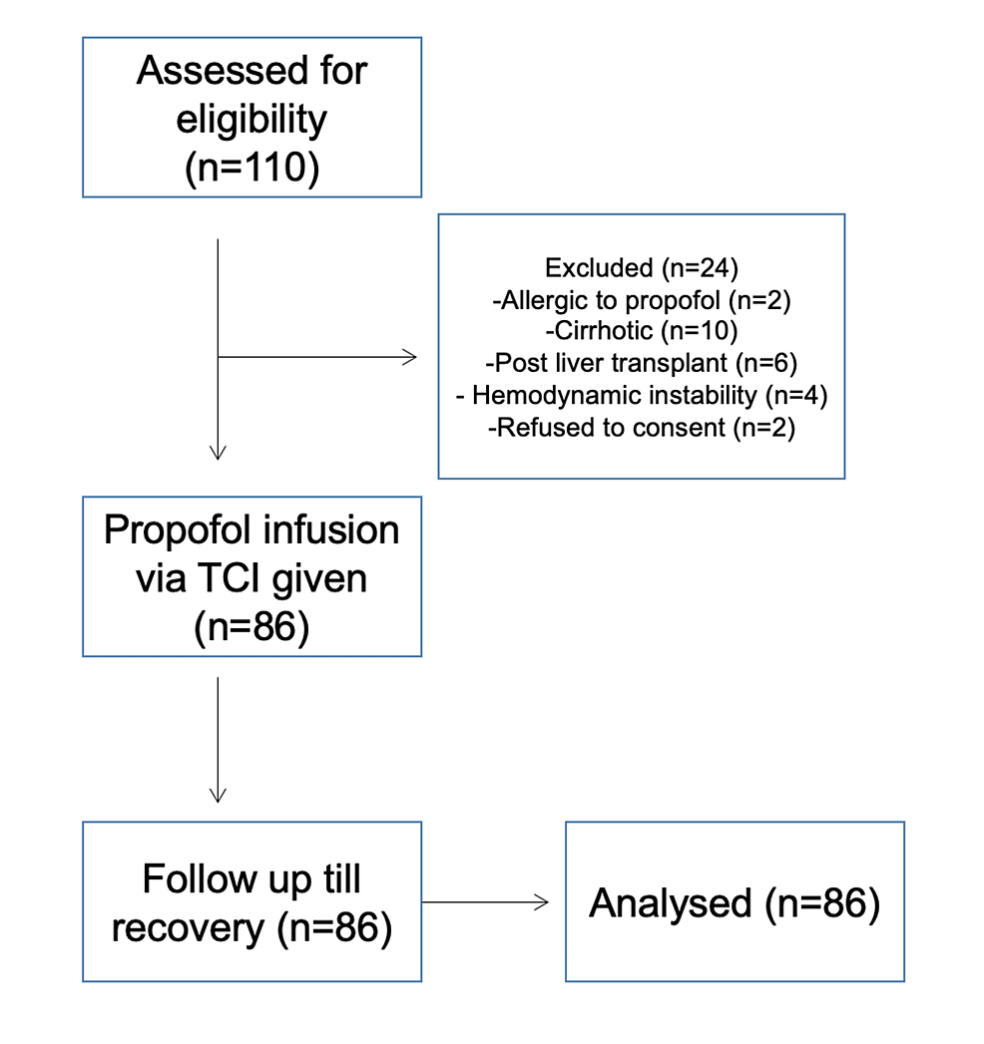
Flow diagram of the interventional study n = number of patients TCI, target control infusion

The patient flow through the trial is illustrated in Figure 1. Of the 110 patients initially assessed for enrolment, 24 were excluded due to various reasons: two for propofol allergy, 10 with cirrhosis, six post-liver transplant, four were hemodynamically unstable, and two refused to participate. Consequently, 86 patients underwent the intervention, and all were followed up until recovery, with no loss to follow-up during the study.

Demographic and clinical variables

**Figure 2:**
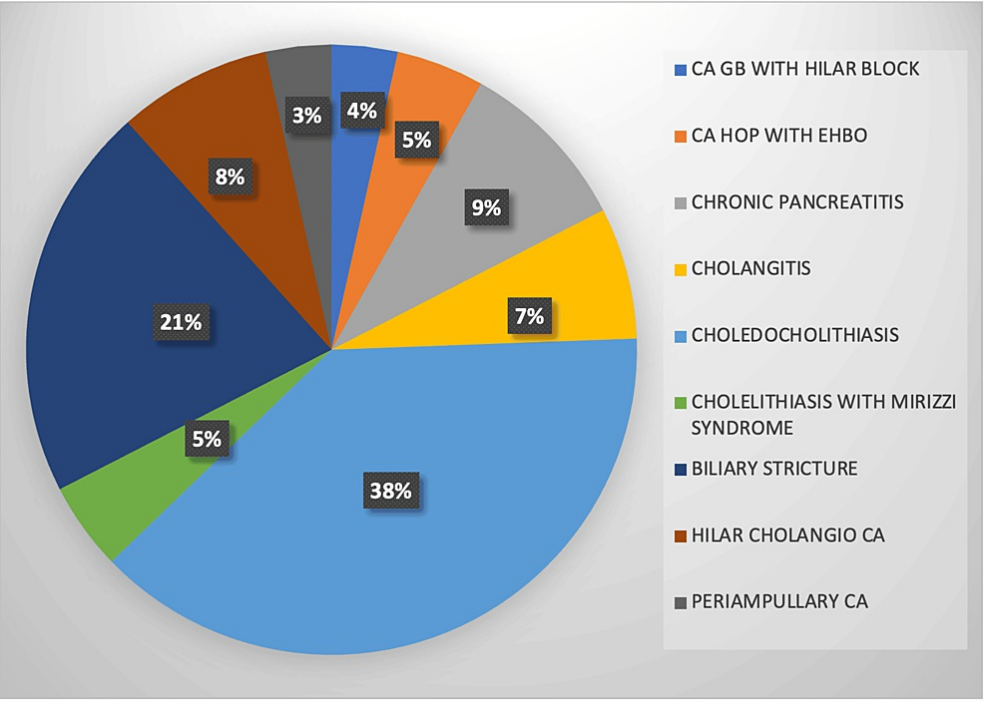
Etiological distribution of the study population Data are represented as percentages (%). CA, carcinoma; EHBO, extrahepatic biliary obstruction; GB, gall bladder; HOP, head of pancreas

Table 1 presents the demographic characteristics of the study population. There was a female preponderance among most patients in the age group of 41-64 years. All patients were evenly distributed between ASA classes I and II. The etiological distribution of patients undergoing ERCP is presented in Figure 2.

**Table 1:** Demographic characteristics of the study population Data are represented as the number of patients (n) and percentage (%). ASA, American Society of Anesthesiologists

Variables	Frequency (n)	Percentage (%)
Male	32	37.2
Female	54	62.5
18-40 years	22	25.6
41-64 years	52	60.5
65-70 years	12	14
ASA class I	43	50
ASA class II	43	50

Study outcomes

**Figure 3:**
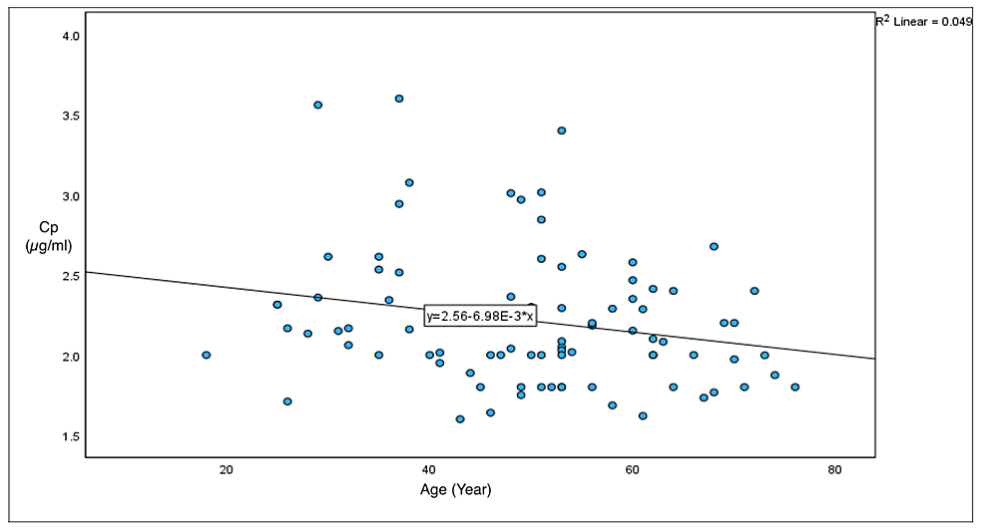
Relationship between age and target Cp of propofol Age is represented in years, and Cp is represented in µg/ml. Cp, plasma concentration

The mean Cp of propofol to maintain a BIS of 60-70 was 2.21 ± 0.42 µg/ml. Age-wise analysis revealed Cp in the 18-40 years group as 2.42 ± 0.48 µg/ml, in the 41-60 years group as 2.16 ± 0.39 µg/ml, and in patients ≥65 years as 2.03 ± 0.28 µg/ml (p = 0.0049), which was statistically significantly different among the age groups. The average time required for the induction of sedation was 4.21 ± 0.68 minutes (min). Recovery and clinical recovery times were reported as medians with IQRs: seven minutes (IQR: 5-10 minutes) and seven minutes (IQR: 5-10 minutes), respectively. Mean propofol consumption through the TCI pump was 6.24 ± 1.98 mg/kg/hr (Table 2). Age had a weekly negative correlation with the target concentration of propofol with a Pearson coefficient of -0.22, i.e., with increasing age, Cp tended to decrease (Figure 3).

**Table 2:** Study outcomes Cp, induction time, and mean propofol consumption are represented as mean ± SD. Recovery time is represented as median with IQR. BIS, bispectral index; Cp, plasma concentration; MOAA/S, Modified Observer’s Assessment of Alertness/Sedation

Variable	Outcome
Cp (µg/ml)	2.21 ± 0.42
Cp in 18-40 years (µg/ml)	2.424 ± 0.48
Cp in 41-64 years (µg/ml)	2.162 ± 0.39
Cp in ≥65 years (µg/ml)	2.035 ± 0.28
Induction time (minutes)	4.21 ± 0.68
Recovery to BIS >80 (minutes)	7 (5-10)
Recovery to MOAA/S ≥5 (minutes)	7 (5-10)
Mean propofol consumption (mg/kg/hr)	6.24 ± 1.98

Side effects

Table 3 illustrates the incidence of various side effects. No major adverse events occurred during the study, 33 (38%) experienced movement during the procedure and required an increase in drug dose. Hypoxia, hypotension, and sinus arrhythmia were observed in one patient each (1.16%). None of the patients reported pain on injection or nausea.

**Table 3:** Incidence of side effects Data are represented as the number of patients (n) and percentage (%).

Side effects	Number of patients	Percentage
Pain on injection	0	0
Transient hypoxia	1	1.16
Hypotension	1	1.16
Respiratory depression	0	0
Patient movement	33	38
Bradycardia	0	0
Tachycardia	0	0
Nausea/vomiting	0	0
Arrhythmia	1	1.16

## Discussion

Using the Marsh pharmacokinetic model for TCI, the present study showed that the mean Cp to maintain a deep level of sedation (BIS of 60-70) during ERCP is 2.21 ± 0.42 µg/ml. Age-wise analysis revealed that the younger patients (18-40 years) require a relatively higher Cp (2.424 ± 0.48) µg/ml compared to the older cohorts. These findings emphasize that the Cp should be individualized based on the patient’s age. The Marsh pharmacokinetic model was chosen due to its superior correlation with both MOAA/S and BIS scores during propofol sedation [10].

A few studies describe the use of TCI of propofol for patients in whom monitored anesthesia care was provided. In a retrospective analysis, Ogawa et al. reported a Cp of 2.2 µg/ml in adults under 70 years undergoing ERCP [11], whereas Cashion and Treston reported a Cp of 1.55 ± 0.54 µg/ml in adults undergoing dental procedures [12]. In both of these studies, BIS was not used to monitor the depth of anesthesia. The only study that used BIS guidance for titrating the TCI rate is by Chhabra et al., who used it in patients undergoing surgery under neuraxial block. They reported a Cp of 1.13 ± 0.17 µg/ml [13]. The use of midazolam by Cashion and Treston and neuraxial block by Chhabra et al. could have contributed to the lower Cp reported by them as compared to the present study. ERCP is a distinctly different procedure that entails heightened gastrointestinal stimulation and, hence, is likely to have a different dose requirement. This underscores the procedural specificity of the sedation requirement.

The average induction time of 4.21 ± 0.68 min indicates the rapid onset of sedation achieved through the TCI approach, aligning with the pharmacokinetic profile of propofol. The median recovery time of seven minutes (IQR: 5-10 minutes) and clinical recovery time of seven minutes (IQR: 5-10 minutes) indicate a relatively swift recovery, enhancing the overall efficiency and safety of the sedation protocol. The recovery times align with those of Chhabra et al. [13], who reported a recovery time of 7.39 ± 3.41 minutes while using TCI in patients undergoing surgery under neuraxial block. These findings suggest that the present TCI approach provides effective sedation with rapid onset and recovery, minimizing the duration of patient discomfort and procedural delays.

BIS guidance for setting the TCI rate is expected to reduce the total propofol requirement. Indeed, the present study showed a mean propofol consumption of 6.24 ± 1.98 mg/kg/h, which is much less than that reported in a study using BIS guidance with a manual infusion pump (8.32 ± 2.65 mg/kg/h) and a manual infusion pump without BIS guidance (11.43 ± 3.85 mg/kg/h) for patients undergoing ERCP [14,15]. This finding demonstrates the efficiency of the dosing strategy used in the present study in maintaining the target sedation level. The optimization of doses in this manner can be beneficial in reducing the recovery times and minimizing the side effects, a finding that may prove to be crucial in high-risk patients subjected to ECRP.

The incidence of side effects was generally low in our study cohort. Patient movement during the procedure, a common challenge during ERCP performed under sedation, occurred in 38% of patients, necessitating an additional bolus dose. This finding aligns with previous reports; Wang et al. documented patient movement in 22.16% of cases during propofol-fentanyl sedation via manual infusion pumps in ERCP [16], while Dhingra et al. reported a higher incidence of 48% under similar sedation protocols [14]. The variability in these rates underscores the challenges of achieving optimal sedation. The present study aimed to identify the target dose of propofol for adequate sedation during ERCP, with an initial target Cp set at 1.2 µg/ml based on Imagawa et al.’s findings [9]. It is conceivable that a higher target concentration of 2.21 µg/ml (as observed in the present study) from the outset may reduce the incidence of patient movement, thereby minimizing the need for additional bolus doses and enhancing procedural stability.

Hypoxia, hypotension, and sinus arrhythmia were observed in a very limited number of patients. Vargo et al., in a study of propofol sedation for ERCP using a manual infusion pump, observed transient hypoxia in 18% of patients, respiratory depression and apnea in 13.33% of patients each, and hypotension in 8% of patients [17]. Wang et al. also reported higher incidences of respiratory depression (12.06%) and hypoxia (7.04%) during propofol-fentanyl sedation via manual infusion pumps in ERCP [16]. Similarly, Dhingra et al., in their study, administered propofol boluses followed by a manual infusion pump for ERCP and observed transient hypoxia in 18%, hypotension in 16%, bradycardia in 4%, and tachycardia in 10% of their patients [15]. In contrast, the present study encountered notably lower incidences, with only 1.16% each for transient hypoxia and hypotension. This difference may be attributed to the steadier Cp maintained by the TCI pump in contrast to manual infusion pumps.

The present study suggests that performing ERCP procedures using propofol via TCI pumps and BIS guidance offers stable hemodynamics, consistent Cps, and fewer complications compared to the conventional method. However, BIS is not readily available in most endoscopy suites. The authors recommend that TCI pumps should replace conventional manual infusion pumps, as the costs of both types of pumps are now similar. They propose setting an initial target Cp of 2-2.2 µg/ml, with the procedure beginning after four to five minutes, with further dosing adjusted based on patient movement and MOAA/S scores. In sicker patients (ASA III and IV), using a TCI pump with a BIS monitor should be prioritized. At the authors’ center, approximately 80 endoscopic procedures are performed daily, including 4-5 ERCPs, with about 30-35% of patients classified as ASA III and IV. Further studies are needed on this cohort, as their requirements may differ from those of the current study population.

There are certain limitations to this study. First, the trial was a single-center study, and further multi-centric, prospective research may be necessary. Second, the cohort of patients included in the study are ASA I and II, and outcomes of class ASA III/IV and elderly patients could not be studied, and further studies need to be conducted on these cohorts of patients. Third, endoscopist satisfaction was not assessed in our study, which could be considered in future investigations.

## Conclusions

Our prospective interventional study sought to address the existing gap in the literature regarding the optimal target Cp of propofol for sedation during ERCP. Utilizing a TCI approach with the Marsh pharmacokinetic model, we successfully identified a mean propofol concentration of 2.21 ± 0.42 µg/ml to maintain a BIS target of 60-70 during ERCP. The age-wise analysis highlighted variations in propofol requirements, emphasizing the importance of individualized dosing strategies considering age-related pharmacokinetic differences.

The TCI approach provides stable hemodynamics, efficient sedation, and minimal complications, offering a valuable contribution to procedural safety and patient comfort.
